# Mobile health to promote physical activity in people post stroke or transient ischemic attack – study protocol for a feasibility randomised controlled trial

**DOI:** 10.1186/s12883-023-03163-0

**Published:** 2023-03-28

**Authors:** Charlotte Thurston, Lucian Bezuidenhout, Sophia Humphries, Sverker Johansson, Lena von Koch, Charlotte K. Häger, Lisa Holmlund, Carl Johan Sundberg, Sara Garcia-Ptacek, Lydia Kwak, Michael Nilsson, Coralie English, David Moulaee Conradsson

**Affiliations:** 1 Department of Neurobiology, Care Sciences and Society, Division of Physiotherapy, Karolinska Institutet, 23100 Stockholm, Sweden; 2 Women’s Health and Allied Health Professionals Theme, Medical Unit Occupational Therapy and Physiotherapy, Karolinska University Hospital, Stockholm, Sweden; 3 Department of Neurobiology, Care Science and Society, Division of Family Medicine and Primary Care, Karolinska Institutet, Stockholm, Sweden; 4 Theme Heart & Vascular and Neuro, Karolinska University Hospital, Stockholm, Sweden; 5 Department of Community Medicine and Rehabilitation - Physiotherapy Section, Umeå University, Umeå, Sweden; 6 Department of Neurobiology, Care Science and Society, Division of Occupational Therapy, Karolinska Institutet, Stockholm, Sweden; 7 Department of Physiology and Pharmacology, Karolinska Institutet, Stockholm, Sweden; 8 Department of Learning, Informatics, Management and Ethics, Karolinska Institutet, Stockholm, Sweden; 9 Department of Neurobiology, Care Sciences and Society, Center for Alzheimer Research, Division of Clinical Geriatrics, Karolinska Institutet, Stockholm, Sweden; 10 Theme Inflammation and Aging, Karolinska University Hospital, Stockholm, Sweden; 11 Institute of Environmental Medicine, Unit of Intervention and Implementation Research for Worker Health, Karolinska Institutet, Stockholm, Sweden; 12 Heart and Stroke Research Program, Hunter Medical Research Institute, Newcastle, NSW Australia; 13 Centre for Research Excellence in Stroke Recovery and Rehabilitation, Florey Institute of Neuroscience and Mental Health, Melbourne, VIC Australia; 14 The Centre for Rehab Innovations (CRI), College of Health, Medicine and Wellbeing, University of Newcastle, Newcastle, Australia; 15 Lee Kong Chian School of Medicine, Nanyang Technological University, Singapore, Singapore

**Keywords:** Complex interventions, Behaviour change, E-Health, Feasibility, Physical exercise, Secondary prevention

## Abstract

**Background:**

Physical activity is essential to improve health and reduce the risk of recurrence of stroke or transient ischemic attack (TIA). Still, people post stroke or TIA are often physically inactive and the availability of physical activity promotion services are often limited. This study builds on an existing Australian telehealth-delivered programme (i-REBOUND– Let’s get moving) which provides support for home-based physical activity for people post stroke or TIA. The aim of this study is to test the feasibility, acceptability, and preliminary effects of a mobile Health (mHealth) version of the i-REBOUND programme for the promotion of physical activity in people post stroke or TIA living in Sweden.

**Methods:**

One hundred and twenty participants with stroke or TIA will be recruited via advertisement. A parallel-group feasibility randomised controlled trial design with a 1:1 allocation ratio to 1) i-REBOUND programme receiving physical exercise and support for sustained engagement in physical activity through behavioural change techniques, or 2) behavioural change techniques for physical activity. Both interventions will proceed for six months and be delivered digitally through a mobile app. The feasibility outcomes (i.e., reach, adherence, safety and fidelity) will be monitored throughout the study. Acceptability will be assessed using the Telehealth Usability Questionnaire and further explored through qualitative interviews with a subset of both study participants and the physiotherapists delivering the intervention. Clinical outcomes on preliminary effects of the intervention will include blood pressure, engagement in physical activity, self-perceived exercise self-efficacy, fatigue, depression, anxiety, stress and health-related quality of life and will be measured at baseline and at 3, 6 and 12 months after the baseline assessments.

**Discussion:**

We hypothesise that the mHealth delivery of the i-REBOUND programme will be feasible and acceptable in people post stroke/TIA living in rural and urban regions of Sweden. The results of this feasibility trial will inform the development of full-scale and appropriately powered trial to test the effects and costs of mHealth delivered physical activity for people after stroke or TIA.

**Trial registration:**

ClinicalTrials.gov Identifier: NCT05111951. Registered November 8, 2021.

## Background

Stroke is largely preventable yet it remains the second-leading cause of death globally and the third-leading cause of death and disability combined [1]. Rates of recurrent stroke or other cardiovascular events following a stroke are high [2, 3], with a cumulative risk of stroke recurrence of 26.4% at 5 years post stroke [2]. Furthermore, individuals who have undergone their first stroke or transient ischaemic attack (TIA) have a sixfold increase in the risk of a recurrent stroke, with secondary events resulting in greater mortality as well as often being both more disabling and costly [4, 5]. TIA is, in addition, a strong predictor of stroke [6] and an independent predictor of long-term mortality [4, 5]. While pharmacological management is crucial in secondary stroke prevention, non-pharmacological interventions focusing on lifestyle modifications (e.g., physical activity and diet) also play an important preventive role [3, 7].

People post stroke or TIA are prone to sedentary lifestyles as sedentary time has been shown to be proportional to increased age [8, 9], with most people experiencing a stroke or TIA at 65 years of age or older [10]. Previous studies have reported that people post stroke spend approximately 30% more time sitting [11] and take less than half the number of steps per day (4078 vs 8338 steps) [12], when compared to healthy individuals. Physical activity has been shown to improve cardiorespiratory fitness and decrease stroke related burdens such as fatigue, depression, and anxiety [13]. Engagement in moderate to vigorous physical activity has shown promising effects on cardiovascular health (e.g., blood pressure reduction) post stroke or TIA [7, 14] with a 40% risk reduction of stroke recurrence 3 years after stroke onset in those who are physically active [15].

Several existing challenges need to be addressed when developing interventions to promote engagement in physical activity post stroke or TIA. First, self-management strategies for physical activity alone have not been shown to increase physical activity post stroke or TIA [14, 16, 17]. Instead, studies have shown that combining supervised exercises with behaviour change techniques for physical activity for more than 4 months to be the most effective in increasing physical activity and improving cardiovascular health [7, 14]. Core behaviour change components of physical activity engagement post stroke or TIA include individual counselling [18], goal setting [19, 20], self-monitoring and structured follow-ups [20] in order to address known barriers for behaviour change. Such barriers are often related to self-efficacy (i.e. the belief a person has in his or her ability to perform a particular behaviour successfully and obtain the intended results) [21], health literacy, and adherence [22]. Second, whilst supervised exercise is often available to people post stroke or TIA, barriers such as rural living and a lack of transportation to facilities which are often located far away hinder access to such services [23].

In Sweden approximately 30,000 people experience a stroke or TIA annually, of which around 20% withstand a recurrent episode [10]. With a population density of 25 people/km^2^ [24] many individuals have limited access to healthcare services with a large variation in the type and availability of stroke rehabilitation observed [10]. One of the challenges for rehabilitation clinics is to provide support for physical activity, particularly for those living remotely [10]. This could be accomplished through mobile health (mHealth), i.e. medical and public health services supported by mobile devices (e.g. mobile phones, patient monitoring devices and applications) [25]; a promising avenue for providing equal and accessible health promotion services to people post stroke or TIA. Despite patients and healthcare professionals reporting high levels of acceptance towards mHealth both globally [26] and locally [27], implementation of mHealth services has been limited during the Covid-19 pandemic in Sweden [27]. mHealth can be beneficially adopted at various points during the patient-clinician therapeutic process from assessment, through to intervention, support, and follow-up [28, 29]. Current low-to-moderate level evidence suggests that remote services (such as mHealth) are no less effective than usual care post stroke with respect to activities of daily living [29, 30], health-related quality of life [29, 30] and depression [29–31]. Despite its potential, the feasibility and long-term effects of mHealth services for specifically promoting physical activity post stroke or TIA remain largely unknown [29, 30]. Furthermore, existing mHealth interventions for people post stroke or TIA use a hybrid approach combining one (or more) physical meetings with mHealth services [29, 30]. Few previous studies have evaluated a completely digital approach for promotion of physical activity (i.e., no physical visits) supported by mHealth post stroke or TIA. To make full use of the potential of mHealth services regarding reach and accessibility, the feasibility of a completely digital approach for promotion of physical activity post stroke or TIA needs to be explored.

**Fig. 1:**
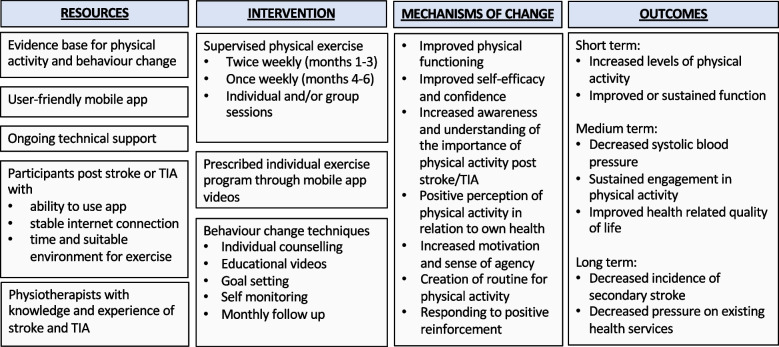
Logic model illustrating intervention theory regarding resources, core intervention components, mechanisms of change, and expected outcomes of this trial

This study is an extension to an existing telehealth-delivered programme (i-REBOUND– Let’s get moving) developed in Australia [32, 33] which provides support for home-based physical exercise and physical activity through behaviour change techniques to people post stroke or TIA via video meetings (i.e., “Zoom”). [34]. The overarching aim of this study is to test the feasibility, acceptability, and preliminary effects of this mHealth version of the i-REBOUND programme for promotion of physical activity in people post stroke or TIA living in Sweden. A logic model has been created to explicitly illustrate intervention theory with regard to resources, core intervention components, mechanisms of change, and expected outcomes of this trial, as shown in Fig. 1. Expected key mechanisms for change in long-term engagement in physical activity are strengthened physical functioning and self-efficacy, positive reinforcement of physical activity, increased awareness of the importance of physical activity for health as well as positive perceptions of engagement in the i-REBOUND programme (e.g., motivation, interest, and creation of routine for sustaining engagement in physical activity) [19].

Specific study objectives are:

- 1. To assess the feasibility of the mHealth version of the i-REBOUND programme regarding reach, protocol and programme adherence, safety and fidelity.
- 2. To explore how people post stroke or TIA experience supervised support for physical activity, and behaviour change support for sustaining engagement in physical activity, through the mHealth version of the i-REBOUND programme.
- 3. To determine the acceptability of the mHealth version of the i-REBOUND programme with respect to mobile app usability and support provided for engaging in physical activity.
- 4. To explore the preliminary effects of the mHealth version of the i-REBOUND programme, compared to the standard approach used in Sweden for general physical activity promotion, on physical activity, blood pressure, exercise self-efficacy, fatigue, depression, anxiety, stress, and health related quality of life.

## Methods

### Design

**Fig. 2:**
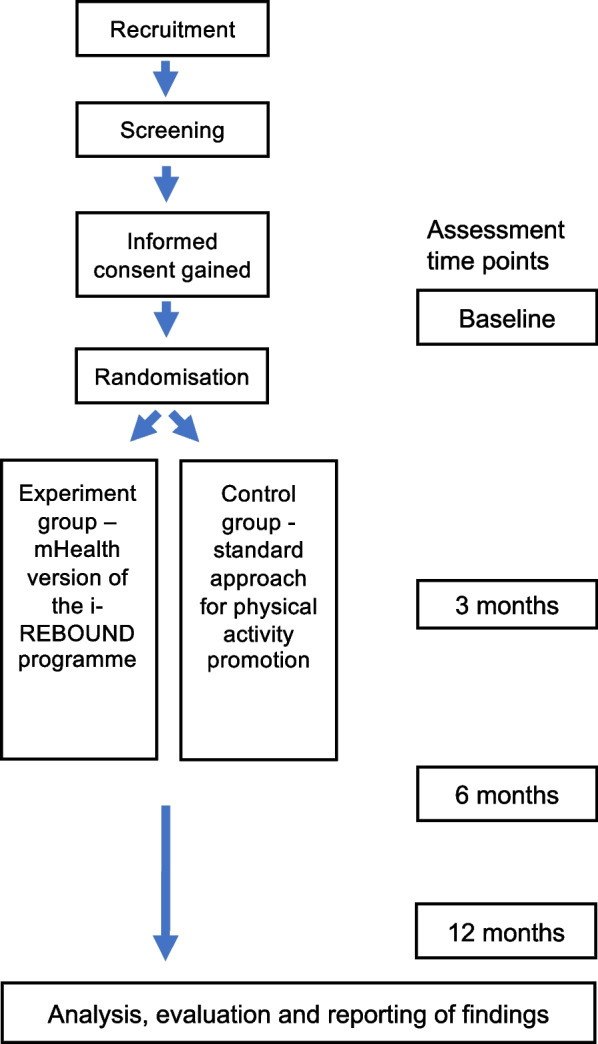
Study flowchart

A prospective two-arm feasibility randomised controlled trial, with an embedded qualitative study, will be conducted in accordance with the CONSORT checklist for feasibility studies [35]. The trial flowchart and design are presented in Fig. 2. The trial is registered at ClinicalTrials.gov (NCT05111951, registered date: 08/11/2021) and has been approved by the Swedish Ethical Review Authority (dnr 2020–05,062, 2021–03,622 and 2022–04,042-02). Written informed consent will be obtained from participants prior to starting the data collection.

### Participants

One hundred and twenty adults diagnosed with a stroke or TIA will be recruited across rural and urban areas of Sweden through advertisements at outpatient clinics, the Karolinska Institutet homepage, patient organisations and social media. The inclusion of 120 study participants builds on our aim to test the feasibility of the mHealth version of the i-REBOUND programme among people post stroke or TIA situated both in urban and rural areas, with variation in disability, exercise habits, physical activity levels, age, gender and technology use. The sample size is in line with recommendations regarding feasibility studies [36].

Inclusion criterion are: (i) between 3 months and 10 years post stroke or TIA at point of study enrolment, (ii) living at home, (iii) with the ability to walk short distances indoors with or without a walking device, (iv) able to use a smartphone, (v) have access to a stable internet connection, and (vi) ability to digitally self-identify using BankID. BankID is a Swedish smartphone and computer application considered to be secure and used by authorities to log into e-services and sign contracts electronically.

Exclusion criterion are: (i) already meeting the recommended physical activity levels of at least 150 min per week of moderate physical activity or at least 75 min per week of vigorous intensity physical activity [37] assessed using the International Physical Activity Questionnaire [38], (ii) severe health conditions (e.g. cardiac conditions, other neurological diseases and severe arthritis), (iii) significant cognitive impairment, neglect or aphasia compromising engagement in the intervention, or iv) enrolled in a concomitant clinical trial or participating in rehabilitation (e.g. aerobic exercises) at the time point of recruitment.

### Study procedures

Study participants will access the respective experimental or control intervention via the STAAR app which is available for iPhone and Android smartphone users. The STAAR app is integrated with a digital clinic; a cloud-based portal accessible through any web browser, enabling therapist-patient communication through chat- and video calls and remote assessment through digital questionnaires. The digital clinic will be used by the physiotherapists delivering the experimental and control interventions, and by the research team to coordinate the trial and to collect data on feasibility, acceptability and clinical outcomes.

Screening for eligibility of study participants will be performed in two steps by members of the research team. First, interested participants will take part in a telephone interview to assess trial eligibility criteria related to stroke or TIA diagnosis, living conditions, ambulation status and mobile phone proficiency. Individuals meeting the inclusion criteria will receive information (through email or regular mail) on how to download, log in and use the video and chat functions in the STAAR app. Subsequently, potential participants will be invited to take part in a video call via the STAAR app including a structured interview regarding the persons experience and ability to download, install, and use different features of the STAAR app (e.g., chat and responding to a questionnaire). During this second interview, potential participants will also be instructed to describe and show where in their home they intend to perform the home-based exercises (if allocated to experimental group) and report on potential barriers and safety aspects related to exercising at home. The video interview will focus primarily on cognitive assessment of potential participants ability to 1) follow instructions, 2) maintain attention during the interview, 3) demonstrate insight into own abilities and limitations, and 4) show insight into safety aspects related to exercising in the home environment. Based on the two-step screening process, alongside a doctor’s certificate confirming diagnosis and ability to engage in moderate to vigorous physical activity, a decision on study inclusion will be made.

### Baseline characteristics

Self-reported demographics (age, sex, comorbidities, level of education, employment status and living situation), date of stroke/TIA, number of previous strokes/TIA, use of mobility device (e.g., walking stick, rollator or wheelchair) and falls history during the previous 6-months will be collected at baseline using digital questionnaires administered through the STAAR app. In addition, self-perceived impact of stroke and lifestyle habits (tobacco, alcohol, physical activity and diet) will be assessed using electronic versions of the Stroke Impact Scale [39] and the screening survey by National Board of Health and Welfare, respectively.

### Randomisation

Following screening and baseline assessment, eligible participants will be randomised to either 1) an experimental group receiving the i-REBOUND programme or 2) a control group receiving the standard approach for physical activity promotion used in Sweden known as ‘Physical Activity on Prescription’ [16]. The randomisation will be performed in blocks of 2 and will be stratified according to mobility status (with/without walking aid) and geographical region (urban/rural areas). The randomisation schedule will be prepared by an independent researcher, and coded into a trial database, ensuring allocation concealment. The researcher responsible for data management and analysis will be blinded to group allocation.

### Interventions

The experimental and control interventions will proceed for six months; an overview of the content of each intervention arm is summarised in Table 1. Each study participant will be allocated to one physiotherapist who will be responsible for their intervention in order to foster the therapist-participant relationship. All therapist-participant interaction will occur through the STAAR app.

**Table 1:**
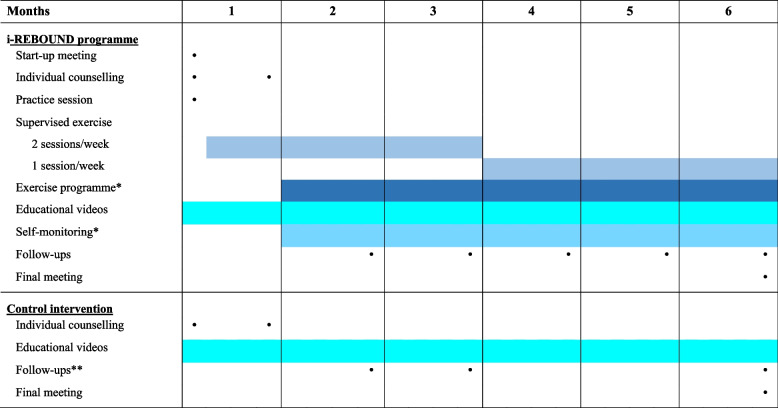
Overview of intervention content for the experimental intervention (i-REBOUND programme) and the control intervention across the 6 months period

Black dots represent individual digital sessions between the physiotherapist and study participants. ^a^Prescription of individual exercise programmes and use of self-monitoring of physical activity are optional parts of the experimental intervention, i.e., will be available to study participants who need extra support for exercise and are interested in monitoring their activity

^b^The 2-month follow-up is optional for the control intervention

All physiotherapists will have previous experience of rehabilitation of people post stroke or TIA and of promoting physical activity in people with neurological diseases through the Swedish Physical Activity on Prescription model [16]. Prior to intervention start, the physiotherapists delivering the experimental arm will participate in two 2-h educational sessions delivered by the research team with a focus on theoretical and practical aspects of the i-REBOUND programme (e.g. supervised exercise, self-monitoring and follow ups). The physiotherapists delivering the control arm will receive one 2-h session on theoretical and practical aspects of the control intervention (e.g., individual counselling and follow ups). An introduction to the STAAR digital clinic focussing on communication channels (e.g., video calls) and delivery of core intervention components will be delivered by a representative from the mobile app developers to both experimental and control group physiotherapists. Ongoing support will be provided by the research team to all physiotherapists via bi-monthly meetings (split according to intervention group) to ensure standardisation of intervention delivery.

## Experiment group – mHealth version of the i-REBOUND programme

The experimental intervention derives from the physical activity component of the i-REBOUND programme [33]. This programme seeks to support uptake of physical exercise to improve function and to support long-term engagement in physical activity through behaviour change techniques. The STAAR app will be used for video calls for individual meetings, supervised exercise sessions and follow-ups, and written communication will be enabled through the chat function.

### Physical exercise

The intervention begins with a *start-up meeting with the physiotherapist (week 1)* including an interview based on the participant's medical history and resources (e.g., physical and social) to engage in home-based exercises and physical activity, with discussion of foreseen barriers. Prior to this meeting, instruction videos about the content and goals of the programme, the STAAR app and safety aspects related to home-based exercises will be made available to study participants in the STAAR app. During week 1 of the intervention, a *practice session of supervised exercise* is performed to foster familiarisation of the practical procedures regarding supervised exercise. This includes approximately 10 min of exercise, information about appropriate placement of the smartphone/tablet during training, safety routines regarding balance support, possible need for assistance from relatives, and routines for assessing health status when the participant should refrain from exercising (e.g., feeling unwell).

*Supervised physical exercise sessions (month 1–6).* The targeted dose will be two exercise sessions per week during months 1–3 and one session per week during months 4–6. The training will include supervised exercises individualised to accommodate stroke related or other deficits in strength, balance, and/or coordination and consist of 5 min warm up followed by 20 min of moderate to vigorous intensity exercise performed in 4 blocks of interval training. Perceived exertion will be rated by participants using the Borg Rating of Perceived Exertion Scale before, midway, and immediately after completing the session [40]. During each training block, participants perform intervals alternating between a more demanding and a less demanding exercise (i.e., active rest). Three exercise interval levels will be implemented (Table 2) and exercise examples include marching with knee lift and arm swing, side stepping, rising from a chair, lunges and squats.

**Table 2:** Content of supervised physical exercise sessions

		**Intervals**^a^			
**Level**	**Description**	**Duration (sec)**	**Number**	**Number of blocks per session**	**Total dose (min)**
1	­ -Standing and sitting exercises ­ -Emphasis on movement quality and stability	High: 30 Low: 30	5	4	20
2	­ -Only standing exercise ­ -Higher pace and greater movement amplitude compared to level 1	High: 45 Low: 15	5	4	20
3	­ -Only standing exercise ­ -Higher pace, greater movement amplitude and increased movement complexity (e.g., combining arm and leg movements) compared to level 2	High: 60 Low: 15	4	4	20

^a^The intervals include switching between a ‘High’ demanding exercise and a ‘Low’ demanding exercise (i.e., active rest)

The training will initially take place individually with the allocated physiotherapist with introduction to group exercise sessions when deemed suitable by the physiotherapist from a safety and logistical perspective (e.g., schedule conflict), and for those who wish to partake in group exercise. Study participants must participate in at least 2 weeks of individual supervised exercise sessions before taking part in group exercise sessions, in which a maximum of 4 participants with a similar mobility status will be included per group. Participants will have the option to interact with each other verbally through a digital waiting room in the STAAR app prior to and during the group training sessions. Training progression will include utilising three different interval durations of exercise (i.e., shorter active rest, see Table 2) and increasing the exercise's speed and amplitude. The physiotherapists will decide when it is appropriate to progress the exercise based on the study participants’ perceived exertion, the quality of movement with regards to postural stability, and safety during the sessions.

*Individual exercise programme (month 2–6)* will be prescribed as instructional videos in the STAAR app according to participants’ needs to further boost engagement in physical exercise. The type of exercises resemble those performed during the supervised sessions and will be prescribed as either strength exercises (e.g., 15 repetitions × 3 sets) or as a block of aerobic exercise (duration 4–8 min). The individuals performing the exercises in these prescribed videos are people of different ages, gender, and disabilities after stroke (e.g., hemiparesis).

### Behaviour change techniques for physical activity

Two *individual counselling sessions (month 1)* will be performed aimed at assessing motivation, exercise preferences and barriers to physical activity, and inform and initiate individual goal setting regarding physical activity. Prior to the first session (week 1), study participants will complete a digital survey on their expectations of participating in the study, perceived barriers to physical activity, and ability and readiness for behaviour change based on a modified version of Patient Goal Priority Questionnaire [41]. Participants’ responses to the survey will guide the discussion between the physiotherapist and study participants regarding motivation, preferences for, and barriers to, physical activity during the first interview. Educational videos regarding physical activity, aerobic and strength exercises, exercise principles (i.e., specificity and progressive overload), physical activity recommendations according to the World Health Organisation [37], and health benefits of physical activity for secondary stroke prevention and health (e.g., blood pressure reduction, mood, sleep) will be available prior to the first interview and throughout the intervention. The educational videos aim to increase study participants’ awareness and understanding about physical activity, exercise and health. The second interview (week 3 or 4) focuses on defining individual physical activity goals [42, 43] with guidance from the physiotherapist, all of which should be SMART. i.e., Specific, Measurable, Achievable, Realistic and Timely [44].

*Self-monitoring of physical activity*. Participants will be encouraged to monitor their physical activity in the STAAR app using an activity diary. The participant chooses which physical activities they want to monitor and days of the week the activities are planned to be carried out. The opportunity of choice is theorised to support the sense of agency and motivation for physical activity and facilitate the process of behaviour change [21]. The activity diary is intended to provide an overview of the activities monitored in a calendar format (week or month view) with the option to receive push notifications as reminders on performing the activity and feedback on percentage of goal fulfilment over time (i.e., per week or month).

*Structured follow-ups* on goal fulfilment and the need to refine the physical activity goals, including support to overcome barriers, as well as individual home exercises programme refinement will be performed once per month through a video meeting (30 min). Prior to the meeting, study participants will complete a digital questionnaire based on a modified version of Patient Goal Priority Questionnaire [41] on perceived barriers to physical activity, goal fulfilment, capacity to perform the targeted exercise or activities and ability to continue performing these activities in the future. Participants responses to this survey combined with self-monitoring of physical activity will be used to guide the discussion during the structured follow-up.

## Control group—standard approach for physical activity promotion

The control group will receive a programme resembling the Swedish model for ‘Physical Activity on Prescription’ [16] which is often used in clinical practice to promote physical activity in people post stroke or TIA in Sweden. As with the experimental group the control intervention includes two individual counselling sessions (intervention week 1 and 3–4) with the goal to assess motivation, exercise preferences and barriers to physical activity, and to define SMART physical activity goals. Prior to the first session, educational videos on physical activity and health (as per experimental group) will be available to the participants. Three structured follow-ups (months 2, 3 and 6) on goal fulfilment and the potential need to revise the physical activity goals will be undertaken. The interviews, goal setting and follow-ups will be performed as described for the experimental group. The therapist-participant communication will take place through video meetings and chat messages via the STAAR app. Support and feedback from the physiotherapist will be limited to the scheduled follow-ups (i.e., no video or chat communication will be available between the scheduled meetings).

## Data collection

### Feasibility

The feasibility outcomes (i.e., reach, adherence, safety and fidelity) are presented in Table 3 and will be monitored from the study launch (i.e., recruitment) to the last follow-up at 12-months post baseline.

**Table 3:** Overview of the feasibility evaluation of the mHealth version of the i-REBOUND programme

Domain	Description	Data collection
***Reach***		
Study recruitment	Proportion of individuals post stroke or TIA screened and deemed eligible for the trial and the proportion of those consenting which are randomised to the trial. Number of study participants requiring assistance to download or access the STAAR app.	Collected by the research team during the recruitment procedures.
Representativeness	Study sample demographics contrasted to national data of the targeted population [13].	Collected by the research team during screening for eligibility and through digital questionnaires administered at baseline.
***Adherence***		
Drop-outs	Number of participants not completing the trial and reasons for withdrawal.	Collected by the research team from point of inclusion until 12-month follow up.
Assessment protocol	Proportion of missing data for clinical outcomes used to assess blood pressure, physical activity, exercise self-efficacy, fatigue, psychosocial wellbeing and health related quality of life.	All clinical outcomes will be self-reported by the participants in the STAAR app at each assessment time point, except for physical activity which will be measured using an activity monitor.
***Safety***		
Adverse events	Number and type of adverse events (including, but not limited to, falls, increase in pain, or hospitalisation).	Reported by either the participants or physiotherapists during the 6-month intervention and recorded by the physiotherapist in the digital clinic.
***Fidelity***		
Supervised exercise	Number of supervised exercise sessions (individual or group) attended with reasons for non-attendance where applicable. Supervised exercise intensity and progression (i.e., changes in the supervised exercise level).	Reported by the physiotherapists in the digital clinic after each supervised exercise session. The Borg RPE scale will be used to assess exercise intensity.
Prescribed exercise	Number of study participants who have been prescribed exercises and prescribed exercise dose (i.e., number of exercises and repetitions).	Information registered automatically in the digital clinic at point of prescription by the physiotherapist.
Behaviour change techniques	Number of study participants taking part in individual counselling and structured follow-ups. Number of goals, goal characteristics, and goal fulfilment across the intervention period.	Recorded by the physiotherapist in the digital clinic after each individual counselling and follow-up session.
Technology usage	Number of times the STAAR app is accessed by study participant and the purpose of accessing (e.g., exercise programme, educational videos and self-monitoring). Number of technical problems including inability to connect to the STAAR app or attending meeting with physiotherapist.	STAAR app access recorded automatically in the digital clinic. Technical problems reported by both participants and physiotherapists to the research team.

### Acceptability

Acceptability of the mHealth version of the i-REBOUND programme will be evaluated using the Telehealth Usability Questionnaire administered immediately post intervention via the STAAR app [45]. Further, a subset of study participants (*n* = 10–15) from the experimental group will be invited to two semi-structured individual interviews early (months 2–3) and late (months 5–6) during the programme for exploration of experiences regarding the mobile app in terms of usability and support, and the physical exercise and behaviour change components of the intervention. Data collection via two separate interviews will enable exploration of similarities and differences in participants’ perceptions and acceptability of mHealth at the different time points. To gain insight into experiences of intervention delivery, interviews will be conducted post-intervention with the physiotherapists responsible for delivering the interventions (*n* = 2–4). All interviews will be conducted via Zoom, audio and video-recorded and transcribed verbatim.

### Clinical outcomes

Clinical outcomes on the preliminary effects of the intervention will be collected at baseline and at 3, 6 and 12 months after the baseline assessments (Table 4). Study participants adherence to the measurement protocol will be monitored by the research team throughout the intervention period with reminders sent to participants through the STAAR app, as required.

**Table 4:** Overview of clinical outcome measures for both experiment and control groups from baseline to one year follow up

Outcome measure	Data collection instrument	Baseline	Month 3	Month 6	Month 12
Physical activity	ActivPal accelerometer	✓	✓	✓	✓
Blood pressure	Omron M7 Intelli IT-AFIB blood pressure monitor	✓	✓	✓	✓
Self-efficacy for exercise	Exercise Self-Efficacy Scale	✓	✓	✓	✓
Balance confidence	Activities-Specific Balance Confidence scale	✓	✓	✓	✓
Walking ability	Generic Walk-12 Scale	✓	✓	✓	✓
Fatigue	Fatigue Severity Scale	✓	✓	✓	✓
Psychosocial wellbeing	Depression Anxiety Stress Scale	✓		✓	✓
Health related quality of life	EuroQol-5 Dimensions	✓		✓	✓

*Physical activity* (steps per day and time spent sedentary, upright, and walking) will be measured using activPAL activity monitors [46]. Monitors will be posted to participants at each assessment point with a pre-paid return envelope. The participants will be asked to wear the device on their non-hemiparetic leg (or dominant leg if no hemiparesis) for 7 consecutive days, after which they will return the device to the research team.

*Systolic and diastolic blood pressure* will be measured using an automated blood pressure monitor (Omron M7 Intelli IT-AFIB) [47] sent to participants prior to baseline measurements. Participants will be instructed to take two recordings of blood pressure on the left upper-arm or the non-affected arm, twice a day (morning and evening) on 7 consecutive days [48] and record their results in the STAAR app. Recorded systolic and diastolic average values will be used for analysis where a minimum of 6 measurements or 3 days have been recorded. We will primarily focus on the evaluation of systolic blood pressure in this study due to the strong linear relationship between systolic blood pressure reduction and risk reduction of recurrent stroke [49].

Digital questionnaires administrated through the STAAR app will be used to assess self-efficacy for exercise, balance confidence, walking ability, fatigue, psychosocial wellbeing, and health related quality of life (see Table 4). The 9-item *Exercise Self-Efficacy Scale* will be used to assess the impact of factors such as weather, mood, and pain on the ability to continue exercising on a three times-per-week basis at moderate intensities for 20 min per session [50]. For each item, participants indicate their confidence to execute the behaviour on a 100-point percentage scale comprised of 10-point increments, ranging from 0% (not at all confident) to 100% (highly confident). Confidence in maintaining balance whilst performing daily activities will be assessed using the *Activities-Specific Balance Confidence scale* which has been specifically developed for use in stroke rehabilitation [51]. This 16-item tool considers various movements (e.g., walking, bending over) in a variety of physical environments (e.g., icy pavements, stairs) and the confidence performing these activities is reported on an 11-point ordinal scale ranging from 0% (“no confidence”) to 100% (“completely confident”). For the Exercise Self-Efficacy Scale and Activities-Specific Balance Confidence scale an average score is calculated (i.e., maximum 100%) and used for analysis. The Swedish version of *Generic Walk-12 Scale,* developed for use specifically for those with neurological conditions will be used to measure self-perceived walking limitations in everyday life during the past two weeks [52]. The first three items of the scale have three response categories (scored 0–2) whereas the remaining nine items have five response categories (scored 0–4). Item scores are summed to a total score with a possible range between 0 and 42 (higher score = more walking difficulties). The 9-item *Fatigue Severity Scale* will be used to evaluate the effects of fatigue on physical everyday activities and life [53]; a nine-item questionnaire with each item scored on a 7-point Likert scale ranging from 1 (‘‘disagree’’) to 7 (‘‘fully agree’’). The mean score of the nine items is presented as a fatigue score. The 21-item *Depression Anxiety Stress Scale* will be used to measure emotional states of depression, anxiety and stress [54]. Each of the three depression and anxiety scales contains 7 items whereby participants rate the extent to which they have experienced symptoms related to depression (e.g., hopelessness, lack of interest and involvement), anxiety (e.g., autonomic arousal, skeletal muscle effects) and stress (e.g., difficulty relaxing, being easily upset, irritable and impatient) over the past week using a 4-point Likert scale. Scores for depression, anxiety and stress are calculated by summing the scores for the relevant items. Health related quality of life will be measured using the *EuroQol-5 Dimensions questionnaire* including five items of different health domains; mobility, self-care, activities of daily living, pain and anxiety/depression, which is scored on 5-point Likert scale (“no problems” to “extreme problems”) and a visual analogue scale (0–100) where the endpoints are labelled “The best health you can imagine” and “The worst health you can imagine” [55].

## Analysis

Baseline characteristics will be presented in numbers and percentages, and in mean and standard deviation or median and interquartile range, depending on the normality of data distributions determined by visual inspection of plots and Shapiro–Wilk tests. Outcomes regarding feasibility (i.e., reach, adherence, safety and fidelity) and acceptability (i.e., Telehealth Usability Questionnaire) will primarily be analysed using descriptive statistics.

For the qualitative analysis of end users’ experiences of the i-REBOUND programme, interview transcripts will be analysed according to thematic analysis [56] following a six-phase dynamic process of data familiarisation, generating semantic and latent codes, actively developing initial themes, reviewing themes, defining and naming themes and producing the report. Inductive analysis will be carried out keeping the themes strongly linked to the data produced during interviews.

Preliminary effects of the mHealth version of the i-REBOUND programme will be analysed using an intention-to-treat and a mixed-effects model to analyse differences in clinical outcomes between the two groups (2 levels: experimental vs control) and over time (4 levels: baseline, and the 3, 6 and 12-months follow-up). Outcomes not meeting the assumptions for analysis using a mixed-effects model will be analysed using non-parametric tests.

## Ethics and dissemination

Collected data will be treated confidentially and only the researchers will have access to the code key. Storage of personal information and registration of databases will follow General Data Protection Regulation laws. Sensitive paper files will be kept in a locked cabinet at the university or digitally on a secured server.

We recognise that people post stroke or TIA may experience cognitive and/or physical impairments, and thereby might have frail health and limited autonomy, potentially posing challenges during study participation. Furthermore, participants may share experiences regarding their disability or life situation which could be distressing for them. The research team and intervention physiotherapists are professionals with competence in how to meet and support people in such situations and in the case that professional help is required information and support for such contacts will be provided. We recognise participants may be disappointed with allocation to the control group, in which case they will be contacted by a member of the research team to discuss further.

Dissemination of study results is planned nationally and internationally via publication of scientific articles and through both poster and oral presentations at conferences.

## Discussion

Our study is a feasibility randomised controlled trial that aims to test the feasibility, acceptability and preliminary effects of the mHealth version of the i-REBOUND programme for promotion of physical activity in people post stroke or TIA across Sweden. This study has the potential to provide new insight and knowledge into the feasibility and acceptance of mHealth for promoting physical activity post stroke or TIA.

Although mHealth holds great potential to improve the accessibility of rehabilitation and health promotion services for people post stroke or TIA [30], several potential operational issues are involved in undertaking a completely digital intervention (i.e., without physical visits). Firstly, relying on technology is prone to technical problems, e.g., due to need for system updates and maintenance, and difficulties managing technology among study participants. Regarding the latter, people post stroke might have cognitive, visual and fine motor impairments affecting their ability to manage technology, e.g., difficulties with reading the font on a screen, orienting the user-interfaces and manual handling of the device [57, 58]. To minimise these risks and facilitate the intervention's reach, the intervention will be delivered using a mobile app developed together with people post stroke or TIA and physiotherapists to foster usability and to meet the needs of people post stroke/TIA [34]. Written and digital information on usability for study participants and physiotherapists delivering the intervention have also been established, as well as routines regarding how to support study participants when technical problems occur. Furthermore, younger age, high level of education and good health status have been associated with greater uptake of new technology for remote services among older adults during the pandemic [59]. This combined with recruitment through advertisement leads to a risk that the present intervention mainly reaches a certain sub-group of people post stroke or TIA. The evaluation of reach and uptake of this intervention is therefore vital for programme feasibility.

This trial investigates a complex mHealth programme with multiple interacting intervention components across a 6-month period. Conducting a large-scale feasibility randomised controlled trial, with an embedded qualitative study, is an important step to capture key uncertainties and understand what needs to be developed or adapted before commencing the full scale randomised controlled trial. Assessment of feasibility and acceptability of the intervention in a Swedish setting is also deemed necessary since the intervention has been developed in Australia [32, 60]. Physiotherapists will be trained in applying the mHealth version of i-REBOUND programme to optimise standardisation, promote adherence to the multifaceted content and ensure optimal quality of intervention delivery. It is also important to consider aspects of the study design (e.g., recruitment, drop-outs and assessment protocol) and safety and fidelity of key intervention components (i.e., physical exercise and behavioural change strategies) which are successfully delivered and which parts of the intervention that are potentially compromised. The transparent reporting should be of benefit to others who wish to replicate the feasibility evaluation of mHealth intervention for the promotion of physical activity post stroke or TIA, whilst avoiding repetition of disadvantageous elements.

## Conclusion

Our mHealth trial addresses a large gap in secondary stroke prevention – accessible and sustainable interventions that enable people with stroke or TIA to be physically active. The results of this feasibility trial will inform the development of full-scaled and appropriately powered trial to test the effects and costs of mHealth delivered physical activity for people post stroke or TIA in Sweden.

## Data Availability

The datasets generated and/or analysed during the present study are not publicly available due to Swedish legislation. Data are available upon reasonable request, and requests for data access can be put to our Research Data Office (rdo@ ki.se) at Karolinska Institutet and will be handled according to the relevant legislation.

## Abbreviation

TIA: Transient ischaemic attack
